# Study design and baseline characteristics of a population-based prospective cohort study of dementia in Japan: the Japan Prospective Studies Collaboration for Aging and Dementia (JPSC-AD)

**DOI:** 10.1186/s12199-020-00903-3

**Published:** 2020-10-31

**Authors:** Toshiharu Ninomiya, Shigeyuki Nakaji, Tetsuya Maeda, Masahito Yamada, Masaru Mimura, Kenji Nakashima, Takaaki Mori, Minoru Takebayashi, Tomoyuki Ohara, Jun Hata, Yoshihiro Kokubo, Kazuhiro Uchida, Yasuyuki Taki, Shuzo Kumagai, Koji Yonemoto, Hisako Yoshida, Kaori Muto, Yukihide Momozawa, Masato Akiyama, Michiaki Kubo, Manabu Ikeda, Shigenobu Kanba, Yutaka Kiyohara, on behalf of the JPSFC-AD Study Group, Toshiharu Ninomiya, Shigeyuki Nakaji, Tetsuya Maeda, Masahito Yamada, Masaru Mimura, Kenji Nakashima, Takaaki Mori, Minoru Takebayashi, Tomoyuki Ohara, Jun Hata, Yoshihiro Kokubo, Kazuhiro Uchida, Yasuyuki Taki, Shuzo Kumagai, Koji Yonemoto, Hisako Yoshida, Kaori Muto, Yukihide Momozawa, Masato Akiyama, Yutaka Kiyohara, Michiaki Kubo, Manabu Ikeda, Shigenobu Kanba, Mao Shibata, Daigo Yoshida, Yoichiro Hirakawa, Takanori Honda, Sanmei Chen, Naoki Hirabayashi, Yoshihiko Furuta, Akane Mihara, Taro Nakazawa, Tomoyuri Ohara, Kazushige Ihara, Koichi Murashita, Kaori Sawada, Songee Jung, Yasuo Terayama, Hisashi Yonezawa, Junko Takahashi, Hiroshi Akasaka, Moeko Noguchi-Shinohara, Kazuo Iwasa, Kenji Sakai, Koji Hayashi, Hidehito Niimura, Ryo Shikimoto, Hisashi Kida, Yoko Eguchi, Yasuyo Fukada, Hisanori Kowa, Kenji Wada, Masafumi Kishi, Taku Yoshida, Hideaki Shimizu, Ayumi Tachibana, Shu-ichi Ueno, Tomohisa Ishikawa, Ryuji Fukuhara, Asuka Koyama, Mamoru Hashimoto, Midori Esaki, Yuji Takano, Yusuke Inoue

**Affiliations:** 1 Department of Epidemiology and Public Health, Graduate School of Medical Sciences, Kyushu University, 3-1-1 Maidashi, Higashi-ku, Fukuoka, 812-8582 Japan; 2 Department of Social Medicine, Graduate School of Medicine, Hirosaki University, Hirosaki, Japan; 3 Division of Neurology and Gerontology, Department of Internal Medicine, School of Medicine, Iwate Medical University, Morioka, Japan; 4 Department of Neurology and Neurobiology of Aging, Kanazawa University Graduate School of Medical Sciences, Kanazawa, Japan; 5 Department of Neuropsychiatry, Keio University School of Medicine, Tokyo, Japan; 6 National Hospital Organization, Matsue Medical Center, Matsue, Japan; 7 Department of Neuropsychiatry, Neuroscience Ehime University Graduate School of Medicine, Ehime, Japan; 8 Department of Neuropsychiatry, Faculty of Life Sciences, Kumamoto University, Kumamoto, Japan; 9 Department of Neuropsychiatry, Graduate School of Medical Science, Kyushu University, Fukuoka, Japan; 10 Department of Preventive Cardiology, National Cerebral and Cardiovascular Center, Suita, Japan; 11 Department of Health Promotion, School of Health and Nutrition Sciences, Nakamura-Gakuen University, Fukuoka, Japan; 12 Department of Nuclear Medicine and Radiology, Institute of Development, Aging and Cancer, Tohoku University, Sendai, Japan; 13 Kumagai Institute of Health Policy, Fukuoka, Japan; 14 Division of Biostatistics, School of Health Sciences, Faculty of Medicine, University of the Ryukyus, Nishihara, Japan; 15 Department of Medical Statistics, Osaka City University Graduate School of Medicine, Osaka, Japan; 16 Department of Public Policy, The Institute of Medical Science, The University of Tokyo, Tokyo, Japan; 17 Laboratory for Genotyping Development, RIKEN Center for Integrative Medical Sciences, Saitama, Japan; 18 Department of Ocular Pathology and Imaging Science, Graduate School of Medical Sciences, Kyushu University, Fukuoka, Japan; 19 Hisayama Research Institute for Lifestyle Diseases, Fukuoka, Japan; 20 Department of Psychiatry, Osaka University Medical School, Suita, Japan

**Keywords:** Dementia, Alzheimer’s disease, Prospective cohort study

## Abstract

**Background:**

The burden of dementia is growing rapidly and has become a medical and social problem in Japan. Prospective cohort studies have been considered an effective methodology to clarify the risk factors and the etiology of dementia. We aimed to perform a large-scale dementia cohort study to elucidate environmental and genetic risk factors for dementia, as well as their interaction.

**Methods:**

The Japan Prospective Studies Collaboration for Aging and Dementia (JPSC-AD) is a multisite, population-based prospective cohort study of dementia, which was designed to enroll approximately 10,000 community-dwelling residents aged 65 years or older from 8 sites in Japan and to follow them up prospectively for at least 5 years. Baseline exposure data, including lifestyles, medical information, diets, physical activities, blood pressure, cognitive function, blood test, brain magnetic resonance imaging (MRI), and DNA samples, were collected with a pre-specified protocol and standardized measurement methods. The primary outcome was the development of dementia and its subtypes. The diagnosis of dementia was adjudicated by an endpoint adjudication committee using standard criteria and clinical information according to the Diagnostic and Statistical Manual of Mental Disorders, 3rd Revised Edition. For brain MRI, three-dimensional acquisition of T1-weighted images was performed. Individual participant data were pooled for data analyses.

**Results:**

The baseline survey was conducted from 2016 to 2018. The follow-up surveys are ongoing. A total of 11,410 individuals aged 65 years or older participated in the study. The mean age was 74.4 years, and 41.9% were male. The prevalence of dementia at baseline was 8.5% in overall participants. However, it was 16.4% among three sites where additional home visit and/or nursing home visit surveys were performed. Approximately two-thirds of dementia cases at baseline were Alzheimer’s disease.

**Conclusions:**

The prospective cohort data from the JPSC-AD will provide valuable insights regarding the risk factors and etiology of dementia as well as for the development of predictive models and diagnostic markers for the future onset of dementia. The findings of this study will improve our understanding of dementia and provide helpful information to establish effective preventive strategies for dementia in Japan.

## Background

Dementia, which is characterized by the impairment of cognitive function, behavior, and the capacity for everyday activities, is widely acknowledged as a public health and social care priority worldwide. According to the World Alzheimer Report 2015, the estimated number of people with dementia worldwide was 46.8 million in 2015, and that number is expected to double to 74.7 million by 2030 [1]. In Japan, an upward trend in the number of patients with dementia is similarly expected along with the aging of the population. A national survey of dementia conducted by the Ministry of Health, Labour and Welfare of Japan found that the prevalence of dementia is 15% among individuals aged 65 and over [2]. Based on this data, the number of patients with dementia in Japan was estimated to have been 4.62 million nationwide in 2012 and is expected to increase further, reaching about 7 million by 2025 [2, 3]. Moreover, epidemiological evidence has shown that the prevalence of Alzheimer’s disease (AD) has been increasing rapidly in Japan for the last 20 years [4]. Therefore, it has become an urgent national issue to establish comprehensive strategies for the prevention and treatment of dementia, particularly AD, as well as for the care of affected individuals.

Epidemiological studies such as prospective cohort studies have been considered an effective methodology to estimate the current status of dementia and to clarify the risk factors and etiology for dementia. Recent prospective cohort studies conducted in Europe and the USA have reported that environmental factors such as diabetes, obesity, smoking, and physical inactivity contribute to the development of dementia [5]. In addition, genetic factors for the development of dementia have also been examined, with the apolipoprotein E (*APOE*) genotype being identified as the most potent genetic risk factor for the development of AD [6, 7]. Moreover, large-scale genome-wide association studies conducted mainly in Europe and the USA have identified several AD-susceptible genes, including the *CR1* and *CLU* genes [7–9]. On the other hand, lifestyles and genetic backgrounds are known to differ among different countries or ethnicities. Therefore, we considered that it would be of value to explore the risk factors for dementia by using the data from a prospective cohort study of community-dwelling, older Japanese populations.

The Japan Prospective Studies Collaboration for Aging and Dementia (JPSC-AD) is an ongoing prospective cohort study for dementia that includes approximately 10,000 older individuals from 8 research sites in Japan with a pre-specified protocol and standardized measurement methods across the research sites. This study aims to explore the genetic and environmental risk factors for dementia and also to examine the gene–environment interaction on the onset of dementia by establishing a large-scale prospective cohort of Japanese.

## Methods

### Study design

**Fig. 1:**
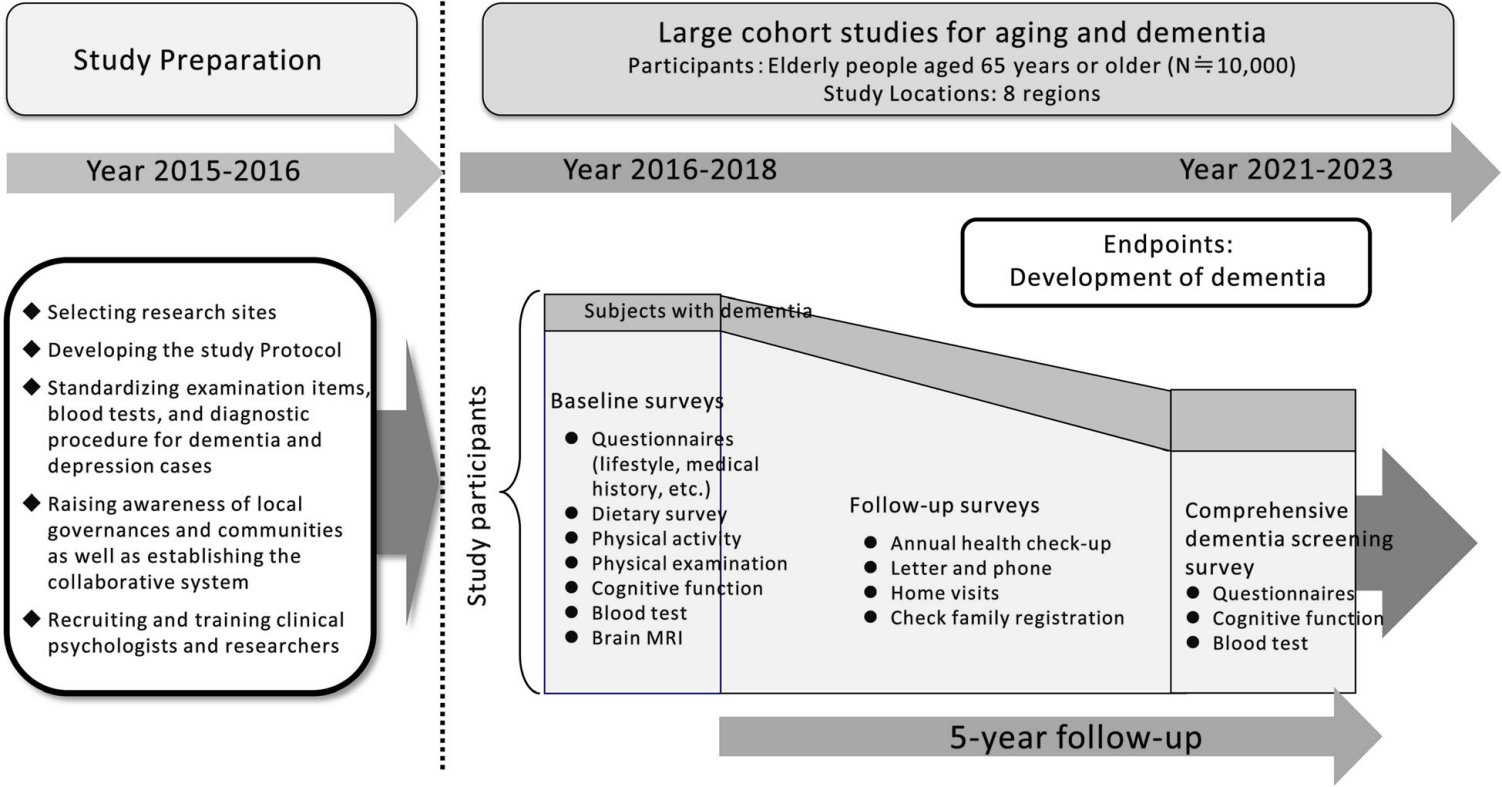
Study design for the Japan Prospective Studies Collaboration for Aging and Dementia (JPSC-AD)

**Fig. 2:**
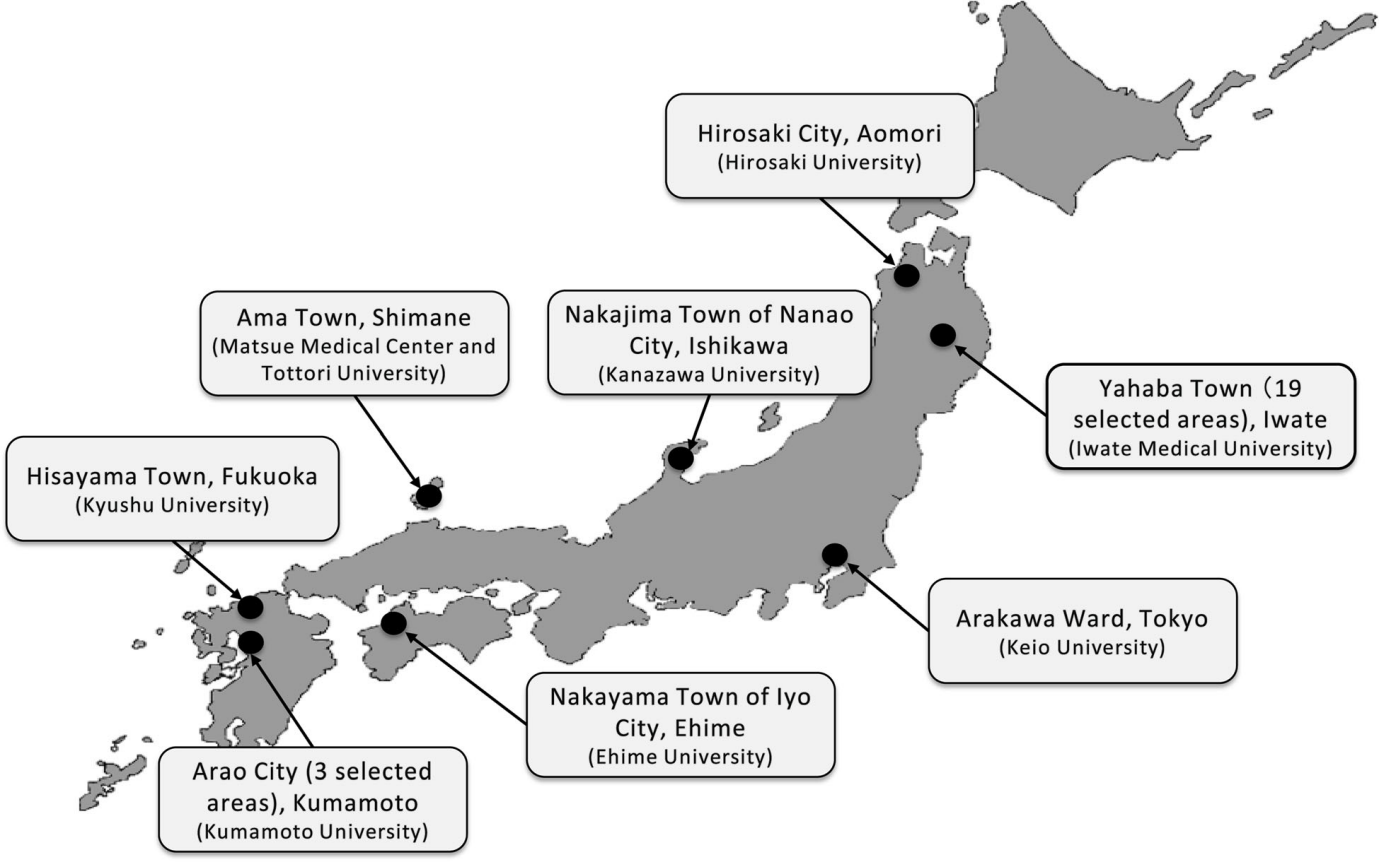
Locations of the 8 research sites for the surveys. The map was downloaded from https://www.start-point.net/maps/material/

The JPSC-AD was designed as a multisite, population-based prospective cohort study for dementia (Fig. 1). At least 10,000 community-dwelling elderly residents aged 65 years and older were surveyed in 8 research sites of Japan as follows: Hirosaki City, Aomori Prefecture (research institute: Hirosaki University); Yahaba Town (19 selected areas), Iwate Prefecture (Iwate Medical University); Nakajima Town of Nanao City, Ishikawa Prefecture (Kanazawa University); Arakawa Ward, Tokyo (Keio University); Ama Town, Shimane Prefecture (Matsue Medical Center and Tottori University); Nakayama Town of Iyo City, Ehime Prefecture (Ehime University); Hisayama Town, Fukuoka Prefecture (Kyushu University); and Arao City (3 selected areas), Kumamoto Prefecture (Kumamoto University) (Fig. 2). First, we pre-specified and standardized the questionnaires, baseline survey items, measurement methods for the blood tests, and diagnostic procedures for dementia across the 8 research sites in 2015, to improve the quality of the collected data. Subsequently, a baseline survey was conducted in 2016–2018. Sampling frames were determined based on the basic resident registers at the initial year of the baseline survey for each research site.

### Study organization

The organization for the JPSC-AD is shown in Supplementary figure 1 and the Acknowledgements. The Steering Committee of this study was organized by the principal investigators of the 8 research institutions. The project and data management were carried out by the central study secretariat set-up at Kyushu University (Center for Cohort Studies, Graduate School of Medicine). An endpoint adjudication committee, a data quality control committee, and a research ethic support committee were also established. A wide-area network data management system was used to manage the survey data; the system was fully equipped with network security (authentication by public key authentication and remote access virtual private network) and always recorded operation logs for data input/output and correction of the information.

### Baseline survey items

The following items were surveyed at baseline using a standardized questionnaire and measurement methods across the 8 research sites.

- Questionnaires: educational history, medical history, medications, smoking habits, alcohol intakes, dietary survey, physical activities, activities of daily living (ADL), functional capacity (instrumental ADL, intellectual activity, social roles), sleeping status, and so on.
- Physical examinations: height, weight, body mass index (BMI), blood pressure, electrocardiogram, grip strength, gait speed
- Neuropsychological testing: cognitive function and depressive symptoms
- Urinalysis: urinary protein, urinary sugar, occult urinary blood, urinary albumin/creatinine ratio
- Blood test: white blood cells, red blood cells, hemoglobin, hematocrit, platelets, total protein, liver function, lipid, uric acid, kidney function, electrolytes, blood glucose, hemoglobin A1C, glycoalbumin, insulin, high-sensitivity C-reactive protein, thyroid function
- Brain magnetic resonance imaging (MRI): three-dimensional acquisition of T1-weighted images (T1WI)
- Preserved blood samples: serum, plasma, and genomic deoxyribonucleic acid (DNA)

Blood pressure was measured three times using an automated sphygmomanometer in the sitting position after at least 5 min of rest, and the mean of the three measurements was calculated. Body height and weight were measured in light clothes without shoes, and the BMI was calculated. Waist circumference was measured at the umbilical level in a standing position. Handgrip strength was measured twice for each hand using a digital strength dynamometer according to the instructions provided by the trained personnel or nurse. The participants were encouraged to exert maximal handgrip strength, and the maximum value between the two hands was used. The participants with pain in their hands and elbows were excluded from the examination of handgrip strength. The usual gait speed was tested twice on the middle 5 m of the course, in which the participants were asked to walk at their usual speed. The faster of the two measurements of the gait speed was used for the analysis. The participants with functional limitations (e.g., walking difficulty or the presence of falling risk) were excluded from the examination of gait speed. The instruments used at each research site to measure blood pressure, electrocardiogram, and handgrip strength are shown in Supplementary table 1.

The detailed measurement methods of blood chemistry are shown in Supplementary table 2. The measurement of blood chemistry was carried out at the central laboratory (LSI Medience Corporation, Tokyo) for all participants using the same equipment in order to eliminate the measurement errors due to the differences in the measuring laboratories. A simple log of the time and temperature from the time of blood collection to sample storage was recorded for the quality control of the collected biological samples. Blood samples (serum and plasma) for preservation were aliquoted and cryopreserved. Genomic DNA was extracted from the whole blood. These samples were stored in − 80 °C deep freezers at the Biological Sample Management Center at Kyushu University.

Depressive symptoms were assessed using the Geriatric Depression Scale (GDS)-short version [10]. Depressive symptoms were defined as a GDS score of ≥ 6 or the current use of antidepressant medication. The subjects with depressive symptoms underwent a second screening survey of depression by using the Mini-International Neuropsychiatric Interview, where depression was diagnosed according to the criteria of the Diagnostic and Statistical Manual of Mental Disorders-fourth edition (DSM-IV) [11, 12].

### Brain MRI examination

The MRI equipment for brain MRI was set with T1WI parameters according to the protocol of brain MRI for the Alzheimer’s Disease Neuroimaging Initiative (ADNI) study [13] at all research sites. In addition, the brain MRI data were standardized by using the MRI Phantom, Human Phantom, and ADNI Phantom to correct geometric distortions among the different pieces of equipment. The volumes of the cortical thickness and area of interest were calculated using image analysis software [14] (FreeSurfer; http://surfer.nmr.mgh.harvard.edu) at the Department of Functional Imaging Medicine, Institute of Development, Aging and Cancer, Tohoku University.

### Diagnosis of dementia

Dementia was diagnosed according to the Diagnostic and Statistical Manual of Mental Disorders, 3rd Revised Edition (DSM-III-R) [15]. The diagnosis of dementia subtypes was made based on the following criteria: the National Institute of Neurological and Communicative Disorders and Stroke and the Alzheimer’s Disease and Related Disorders Association criteria (NINCDS-ADRDA) [16] for AD, the National Institute of Neurological Disorders and Stroke-Association International pour la Recherche et l’Enseignement en Neurosciences criteria (NINDS-AIREN) [17] for vascular dementia (VaD), and the Fourth Consensus Report of the Dementia with Lewy Bodies (DLB) Consortium [18] for DLB. Petersen’s criteria were used for the diagnosis of mild cognitive impairment (MCI) [19].

**Fig. 3:**
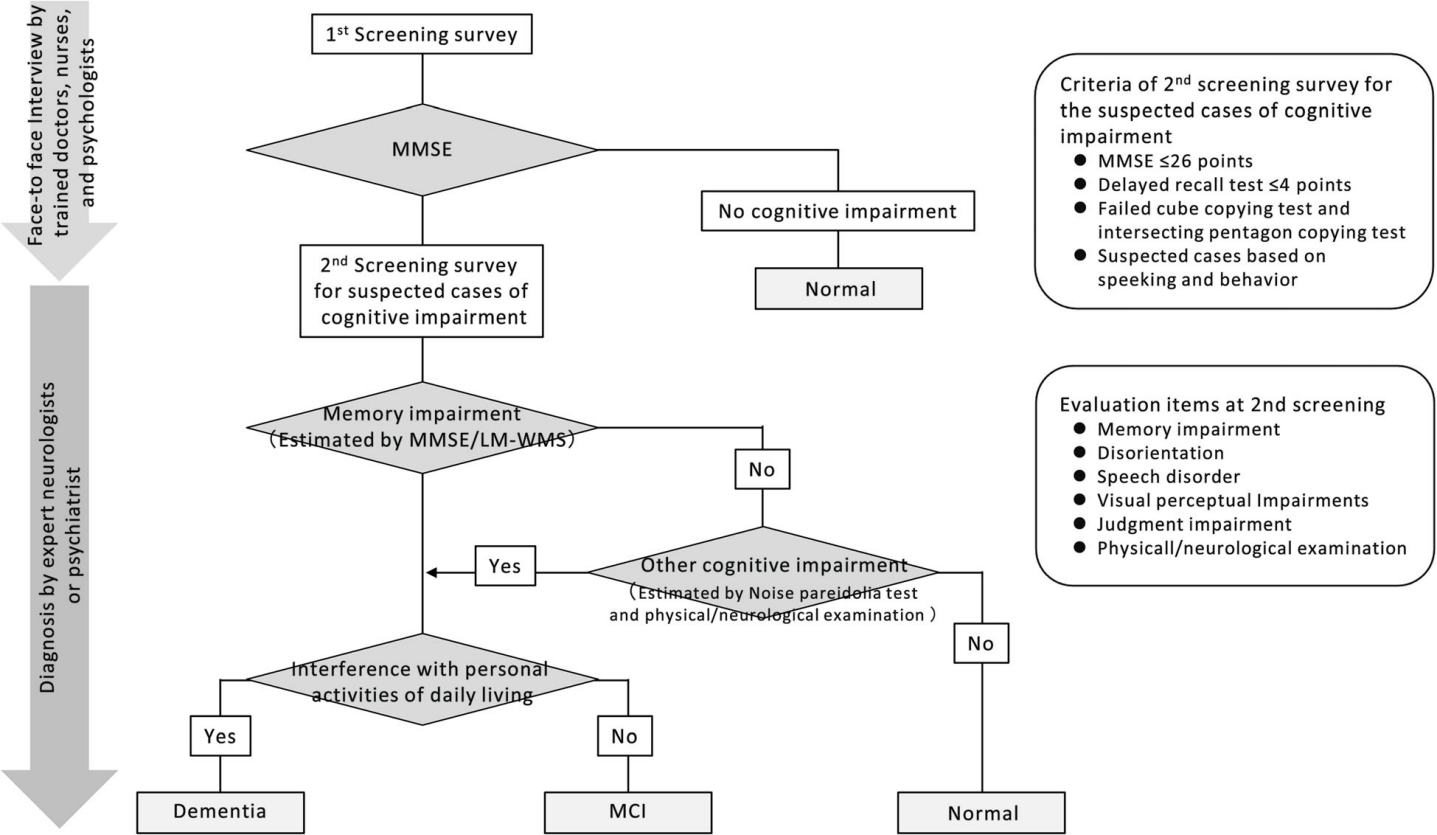
Process of the diagnosis for cognitive impairment. LM-WMS, the delayed recall of the Logical Memory IIA subscale of the Wechsler Memory Scale-Revised; MCI, mild cognitive impairment; MMSE, mini-mental state examination

The diagnosis of dementia was made using a two-step diagnostic system, which was standardized among the 8 research sites (Fig. 3). First, an interview survey for the screening of cognitive function was conducted by trained doctors, public health nurses, nurses, and clinical psychologists using the Mini-Mental State Examination (MMSE) [20] as the first screening survey. The subjects who met the following criteria underwent the second screening survey for the suspected cases of cognitive impairment: (1) MMSE ≤ 26 points, (2) score of ≤ 4 of a total possible 6 points on the delayed recall component of the MMSE (i.e., 3 questions, each scored 2 points if answered correctly without a hint, 1 point if answered correctly with a hint, and 0 points if answered incorrectly), (3) failed intersecting pentagon-copying component in the MMSE and/or cube-copying test [21], and (4) suspected cases based on the manner of speaking and behavior. In the second screening survey, the presence of cognitive impairment (i.e., MCI or dementia) and dementia subtypes was determined by expert psychiatrists or neurologists based on the physical and neurological examinations, including the delayed recall test of the logical memory IIA subscale of the Wechsler Memory Scale-Revised [22] and the Pareidolia test [23] the information from the patient, interviews with family members and attending physicians, medical records, and brain imaging. For the delayed recall test of the Wechsler Memory Scale-Revised, the cut-off scores of any cognitive impairment were selected according to the education levels as follows: ≤ 8 points for 16 years of education, ≤ 4 points for 8–15 years, and ≤ 2 points for 0–7 years [22].

To standardize the accuracy in the diagnosis of cognitive impairment across the 8 research sites, all cases of cognitive impairment were adjudicated by independent evaluators who were expert psychiatrists or neurologists at the different recruitment sites by reviewing the collected clinical information. If the diagnosis of the specialists in each region and the members of the endpoint adjudication committee were in agreement, the diagnosis was confirmed; if not, a meeting of the endpoint adjudication committee was held, and the diagnosis was confirmed through discussion.

### Outcomes of interest

The primary outcome of this study is the development of dementia and its subtypes during the follow-up period. As alternative outcomes, the following information will also be collected during the follow-up period: (1) the changes in cognitive function, (2) the development of cardiovascular disease, including stroke and coronary heart disease, (3) all-cause and cause-specific deaths, and (4) the onset of depressive symptoms. The information at baseline surveys—namely, the presence of dementia and depression, and the brain MRI results—will be used in cross-sectional analyses.

### Follow-up surveys

The follow-up surveys for participants are ongoing. Health check-ups for the study participants have been repeated every 1–2 years to obtain information on the health status of participants and the development of the outcomes of interest. Letter or telephone surveys or home visits have been conducted for individuals who did not undergo a health check-up or complete a dementia survey and who moved away from the vicinity of research sites. In addition, every 5–6 years, a comprehensive survey of dementia will be repeated in the same way as the baseline dementia survey in order to minimize missed cases of dementia. Further, we will collect the death information from the Ministry of Health, Labour and Welfare’s Vital Statistics. When the development of outcomes is suspected, the participants or their family members will be interviewed, and their detailed clinical information will be collected from hospitals or clinics whenever possible.

### Statistical analysis

The required sample size was calculated as 9850 participants using a log-rank test with 80% power and a two-tailed significance level of 5% to detect a hazard ratio of 1.50 on the development of AD for 5 years, under the assumption of a frequency of exposure of 10% and a 5-year incidence rate of AD of 5% [4]. The sample size permitted a loss of 15% of participants to follow-up. Therefore, the required number of enrolled participants in this study was 10,000 or more.

For the baseline characteristics of participants, the values were shown as the mean (standard deviation), median (interquartile range), or frequency, as appropriate. Subjects with missing values were excluded from the analysis for each relevant variable. All statistical analyses were performed with the SAS statistical software program, version 9.4 (SAS Institute Inc., Cary, NC, USA).

## Results

The baseline survey was conducted from 2016 to 2018. A total of 11,957 community residents in 8 research sites consented to participate in the study, of which 11,410 individuals were aged 65 years or older. Full community surveys were conducted at 6 rural sites; at each of these sites, all residents aged 65 years or older were recruited based on the basic resident registers at the initial year of the baseline survey and were encouraged to participate in the surveys. As a consequence, 8030 individuals aged 65 years or older (participation rate, 65% of the total residents of this age group across the 6 sites) consented to participate in these surveys: Yahaba Town (*n* = 962), Nakajima Town (*n* = 2128), Ama Town (*n* = 722), Nakayama Town (*n* = 927), Hisayama Town (*n* = 1714), and Arao City (*n* = 1577). The remaining 3380 individuals were selected by a simple random sampling (Arakawa Ward, *n* = 1099) and a voluntary response sampling (Hirosaki City, *n* = 2281) at the 2 sites with larger populations. The recruited residents visited the research facilities (e.g., the health centers, clinics, or hospitals) for the baseline survey, MRI scans, and blood samplings.

**Fig. 4:**
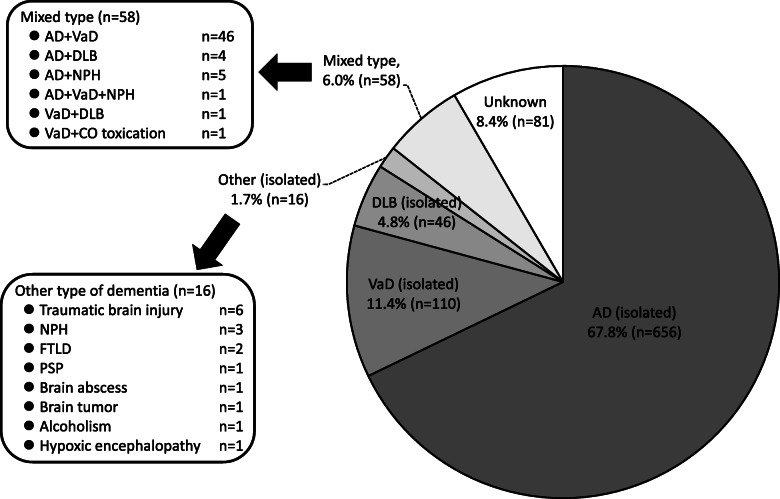
Frequencies of dementia subtypes at baseline. AD, Alzheimer’s disease; VaD, vascular dementia; DLB, dementia with Lewy bodies; NPH, normal pressure hydrocephalus; CO, carbon monoxide; FTLD, frontotemporal lobar degeneration; PSP, progressive supranuclear palsy

The baseline characteristics of the study participants are shown in Table 1. The mean age of participants was 74.4 (standard deviation [SD], 7.2) years. The frequency of men was 41.9%. The crude prevalences of dementia and MCI at baseline were 8.5% and 17.0%, respectively (Table 2). The age-specific prevalence of dementia increased with age in the overall participants, whereas the highest age-specific prevalence of MCI was observed in the participants aged 80–84 years. The same was true in both sexes. The prevalence of dementia was higher in women than in men (6.6% for men vs. 9.8% for women), but men had a higher prevalence of MCI than women (19.9% vs. 14.9%). With regard to dementia subtypes, approximately two-thirds (67.8%) of dementia cases at baseline were cases of AD (isolated type), which was the most frequent subtype of dementia, followed by VaD (11.4%), and DLB (4.8%) (Fig. 4). The frequency of the mixed type of dementia was 6.0%, the primary subtype of which was a combination of AD and VaD.

**Table 1:** Baseline characteristics of the study participants aged ≥ 65 years (*n* = 11,410)

Variables	Mean, median, or frequency^a^	% of missing value
**Demographic factors and lifestyles**		
Age, years	74.4 (7.2)	0.0
Men, %	41.9	0.0
Education ≤ 9 years, %	35.5	1.8
Current smoker, %	8.2	2.3
Current drinker, %	40.2	2.4
Regular exercise^b^, %	40.9	9.4
**Physical examinations**		
Systolic blood pressure, mmHg	139.8 (18.9)	9.0
Diastolic blood pressure, mmHg	78.1 (11.5)	9.0
BMI, kg/m^2^	23.3 (3.4)	8.7
BMI category, %		
< 18.5 kg/m^2^	5.9	
18.5–24.9 kg/m^2^	66.4	
≥ 25.0 kg/m^2^	27.8	
Waist circumference (at umbilicus), cm	85.3 (9.3)	13.5
Electrocardiogram abnormalities^c^, %	14.1	12.8
**Comorbidities and medications**		
History of cardiovascular disease^d^, %	11.8	0.5
History of cancer, %	13.3	0.4
Hypertension^e)^, %	75.0	5.1
Diabetes mellitus^f^, %	18.4	10.5
Use of antihypertensive agents, %	51.2	1.9
Use of glucose-lowering agents (including insulin therapy), %	12.9	2.0
Use of lipid-modifying agents, %	29.0	2.4
**Blood test**		
White blood cells, × 10^9^/L	5.7 (2.4)	11.6
Red blood cells, × 10^12^/L	4.4 (0.5)	11.6
Hemoglobin, g/L	136.8 (14.6)	11.6
Hematocrit, %	42.5 (4.4)	11.6
Platelet, × 10^9^/L	230.2 (62.3)	11.7
Serum total protein, g/L	73.8 (4.9)	11.6
Serum albumin, g/L	43.1 (3.3)	11.6
Serum urea nitrogen, mmol/L	6.1 (1.9)	11.6
Serum creatinine, μmol/L	63.6 (53.9–76.9)	11.6
Estimated GFR^g^, ml/min/1.73m^2^	67.8 (11.8)	11.6
Serum uric acid, μmol/L	305.7 (77.4)	11.6
Serum total bilirubin, μmol/L	12.0 (10.3–15.4)	11.6
Serum aspartate transaminase, U/L	23 (20–28)	11.6
Serum alanine transaminase, U/L	17 (13–23)	11.6
Serum alkaline phosphatase, U/L	232 (192–281)	11.6
Serum lactate dehydrogenase, U/L	203 (181–227)	11.6
Serum γ-glutamyl transferase, U/L	22 (16–35)	11.6
Serum total cholesterol, mmol/L	5.3 (0.9)	11.6
Serum LDL cholesterol, mmol/L	3.0 (0.8)	11.6
Serum HDL cholesterol, mmol/L	1.6 (0.4)	11.6
Fasting serum triglycerides, mmol/L	1.0 (0.8–1.4)	61.0
Fasting blood glucose, mmol/L	5.3 (1.1)	61.0
Fasting serum insulin, pmol/L	28.8 (21.0-40.8)	61.3
Hemoglobin A1c (NGSP), %	5.8 (0.7)	11.6
Serum glycated albumin, %	15.5 (2.6)	12.7
Serum sodium, mmol/L	141.9 (2.1)	11.7
Serum potassium, mmol /L	4.3 (0.6)	11.6
Serum high-sensitivity CRP, mg/L	0.48 (0.23–1.03)	11.6
Serum free thyroxine (T_4_), pmol/L	14.9 (2.3)	11.6
Serum thyroid stimulating hormone, μIU/mL	1.90 (1.27–2.88)	11.6
**Urine test**		
Proteinuria (dipstick ≥ 1+ or Uprot ≥30 mg/dL), %	7.3	13.9
Urinary albumin:creatinine ratio, mg/gCr	11.6 (6.0–29.3)	13.7
**ADL and sleep**		
ADL disability (Barthel index ≤ 95), %	12.2	1.4
Functional capacity impairment (TMIG-IC ≤ 12), %	43.1	2.3
Maximal handgrip strength, kg	27.0 (8.7)	6.0
Usual speed 5-m walking time, s	4.0 (1.4)	21.1
Sleeping time, h	7 (6–8)	8.8
**Cognitive function**		
Mini-Mental State Examination, points	28 (25–29)	3.4
Mild cognitive impairment, %	17.0	0.1
Dementia, %	8.5	0.1
**Depressive status**		
Geriatric depression scale, points	2 (1–4)	4.4
Depressive symptoms (geriatric depression scale ≥ 6 points), %	16.1	4.4
Depression, %	1.2	0.1

Note. Conversion factors for units were as follows: hemoglobin, serum total protein, and serum albumin in g/dL to g/L, × 10; serum urea nitrogen in mg/dL to mmol/L, × 0.357; serum creatinine in mg/dL to μmol/L, × 88.4; serum uric acid in mg/dL to μmol/L, × 59.48; serum total bilirubin in mg/dL to μmol/L, × 17.1; serum cholesterol (total, LDL, and HDL) in mg/dL to mmol/L, × 0.02586; serum triglycerides in mg/dL to mmol/L, × 0.01129; blood glucose in mg/dL to mmol/L, × 0.05551; serum insulin in μU/mL to pmol/L, × 6.00; serum free thyroxine in ng/dL to pmol/L, x 12.87

*ADL* activities of daily living, *BMI* body mass index, *CRP* C-reactive protein, *GFR* glomerular filtration rate, *HDL* high density lipoprotein, *LDL* low-density lipoprotein, *NGSP* National Glycohemoglobin Standardization Program, *TMIG-IC* Tokyo Metropolitan Institute of Gerontology Index of Competence, *Uprot* urinary protein concentration

^a^Values are shown as the mean (standard deviation), median (interquartile range), or frequency, as appropriate. Subjects with missing values were excluded from the analysis for each relevant variable

^b^Regular exercise was defined as any physical activity performed for at least 30 min twice per week over the most recent year or longer

^c^Electrocardiogram abnormalities were defined as the presence of left ventricular hypertrophy, ST depression, and/or atrial fibrillation

^d^Cardiovascular disease was defined as stroke, coronary heart disease, and/or coronary intervention

^e^Hypertension was defined as blood pressure ≥ 140/90 mmHg and/or use of antihypertensive agents

^f^Diabetes was defined as fasting blood glucose ≥ 126 mg/dL, casual blood glucose ≥ 200 mg/dL, hemoglobin A1c ≥ 6.5%, and/or use of glucose-lowering agents

^g^Estimated GFR was calculated by using the Japanese coefficient modified CKD-EPI (Chronic Kidney Disease Epidemiology Collaboration) equation

**Table 2:** Age-specific prevalence of dementia and mild cognitive impairment at baseline among overall participants and by sex

Age group, years	Number of subjects	Number of cases of dementia (prevalence^a^)	Number of cases of MCI (prevalence^a^)	Number of subjects with missing data (%)^a^
**Overall**				
65–69	3728	33 (0.9%)	314 (8.4%)	2 (0.05%)
70–74	2758	65 (2.4%)	364 (13.2%)	4 (0.15%)
75–79	2218	114 (5.1%)	494 (22.3%)	2 (0.09%)
80–84	1462	231 (15.8%)	441 (30.2%)	1 (0.07%)
85–89	816	279 (34.2%)	229 (28.1%)	3 (0.37%)
≥ 90	428	245 (57.2%)	92 (21.5%)	0 (0.00%)
**Total**	**11,410**	**967 (8.5%)**	**1934 (17.0%)**	12 (0.11%)
**Men**				
65–69	1648	15 (0.9%)	198 (12.0%)	2 (0.12%)
70–74	1209	31 (2.6%)	205 (17.0%)	1 (0.08%)
75–79	936	43 (4.6%)	242 (25.9%)	2 (0.21%)
80–84	590	90 (15.3%)	186 (31.5%)	0 (0.00%)
85–89	283	83 (29.3%)	85 (30.0%)	1 (0.35%)
≥ 90	116	53 (45.7%)	34 (29.3%)	0 (0.00%)
**Total**	**4782**	**315 (6.6%)**	**950 (19.9%)**	6 (0.13%)
**Women**				
65–69	2080	18 (0.9%)	116 (5.6%)	0 (0.00%)
70–74	1549	34 (2.2%)	159 (10.3%)	3 (0.19%)
75–79	1282	71 (5.5%)	252 (19.7%)	0 (0.00%)
80–84	872	141 (16.2%)	255 (29.2%)	1 (0.11%)
85–89	533	196 (36.8%)	144 (27.0%)	2 (0.38%)
≥ 90	312	192 (61.5%)	58 (18.6%)	0 (0.00%)
**Total**	**6628**	**652 (9.8%)**	**984 (14.9%)**	6 (0.09%)

*MCI* mild cognitive impairment

^a^For the calculation of prevalence, subjects with missing cognitive function data were assigned neither to the category of dementia nor the category of MCI

^b^Values are shown as the number (percentage) of subjects with missing cognitive function data

In addition, we performed a sensitivity analysis for the prevalence of dementia among 5257 subjects (participation rate of sampling frames, 85%) at 3 research sites (Nakajima Town, Nakayama Town, and Hisayama Town), where the surveys by home visit or by visits to nursing homes or other facilities (e.g., long-stay hospitals) were conducted in addition to the survey at the research facilities. For the home visit, we contacted the participants or their surrogates (e.g., family members) through public health nurses and obtained the permission to visit. For the visits to nursing homes or other facilities, we obtained permission to conduct the survey from the directors of each site and the participants (or their surrogates). Among 5257 subjects, 1555 subjects (29.5%) were surveyed by the home visit, and 463 subjects (8.8%) were surveyed by visiting a nursing home or other facilities. The baseline characteristics of these subjects are shown in Supplementary table 3. The crude prevalence was 16.4%, as shown in Supplementary table 4.

## Discussion

JPSC-AD is a large-scale population-based prospective cohort study for dementia that includes more than 10,000 older individuals from 8 research sites in Japan. The quality-controlled data of exposure and outcomes were collected with a pre-specified protocol and standardized measurement methods across the research sites. In addition, a number of community-based brain MRI data and DNA samples in the elderly are also available for the analyses. The follow-up surveys are ongoing.

The risk of dementia has been considered to be affected by environmental risk factors (e.g., hypertension, smoking, diabetes, physical inactivity) and genetic risk factors (e.g., APOE genotypes). Currently, however, there is limited evidence for establishing effective preventive interventions in order to reduce dementia risk. The gold-standard methodology for clarifying causality is randomized controlled trials, but it is hard to perform intervention trials over a sufficiently long period of time to analyze the efficacy of interventions for some of the risk factors related to the development of dementia, such as educational level, sleeping, and certain genotypes. Therefore, the findings derived from observational studies, especially prospective cohort studies, are valuable to establish possible preventive measures [24]. In Japan, several epidemiological studies for dementia have been conducted [4, 25–28], but their sample sizes have generally been too small for detailed analyses of the influence of the environmental and genomic factors on the development of dementia. Meta-analyses can be useful and informative in integrating research findings across studies with limited sample size. However, meta-analyses with data from pre-existing studies have some unavoidable limitations, such as unreliability in the findings due to the heterogeneity of study design, study quality, measurement methods, and statistical analysis across the included studies [29]. Such limitations can be improved by pooling the data of individual participants collected with standardized measurement methods of exposures and outcomes across the included studies. This was the underlying concept in launching the JPSC-AD.

In the present study, the crude prevalence of dementia was 8.5% at baseline, which was lower than previously reported [2]. The main focus of this study was to elucidate the risk factors for dementia while incorporating the brain MRI data and genomic data, rather than to estimate the prevalence of dementia. Thus, the baseline survey in the present study was mainly conducted among participants who visited the research facilities (e.g., the health centers, clinics, or hospitals) to obtain detailed baseline information including brain MRI data and blood samplings. Hence, the prevalence of dementia at the baseline in the overall participants from 8 sites is likely to be underestimated. Meanwhile, the sensitivity analysis revealed that the crude prevalence was 16.4% among participants from three sites with a high participation rate, where additional surveys by home visit or visits to nursing homes or other facilities were conducted in addition to the survey in the research facilities. This value was comparable to those in the previous reports [2].

In the present study, subjects with depressive symptom were screened by using the GDS score, and subjects with depression were diagnosed according to the criteria of the DSM-IV. Consequently, the percentages of subjects with depressive symptoms and depression were 16.1% and 1.2%, respectively. The previous epidemiological studies reported that the prevalence of subjects with depressive symptoms diagnosed by psychological tests (e.g., GDS score) was about 10–30% [30, 31] which was comparable to the prevalence in our present study. On the other hand, there have been few epidemiological studies addressing the prevalence of depression diagnosed according to the criteria of DSM-IV. Our findings will be valuable in this regard.

## Conclusions

This study is expected to promote identification of the risk factors and the etiology for dementia and advance the development of predictive models and diagnostic markers for developing future dementia. The findings of this study will contribute to the establishment of preventive strategies according to the individual risk of dementia and improve the health, medical care, and welfare of the general Japanese population.

## Supplementary information

**Additional file 1: Table S1.** Instruments to measure blood pressure, electrocardiogram, and handgrip strength. **Table S2.** Measurement methods by item. **Table S3.** The baseline characteristics for 5,257 subjects at three research sites (Nakajima Town, Nakayama Town, and Hisayama Town). **Table S4.** Age-specific prevalence of dementia at baseline among 5,257 subjects at three research sites (Nakajima Town, Nakayama Town, and Hisayama Towna); participation rate: 85%), where surveys by home visit or visits to nursing homes or other facilities (e.g., long-stay hospitals) were conducted in addition to the surveys at the health centres or hospital. **Figure S1.** Organization of the Japan Prospective Studies Collaboration for Aging and Dementia (JPSC-AD).

## Data Availability

The datasets generated and/or analyzed during the current study are not publicly available due to restrictions included in the informed consent of research participants. However, data are available from the authors upon reasonable request and with the permission of the Japan Agency for Medical Research and Development.

## Abbreviations

AD: Alzheimer’s disease
ADL: Activities of daily living
ADNI: Alzheimer’s Disease Neuroimaging Initiative
APOE: Apolipoprotein E
BMI: Body mass index
DLB: Dementia with Lewy bodies
DNA: Deoxyribonucleic acid
DSM-III-R: Diagnostic and Statistical Manual of Mental Disorders, 3rd Revised Edition
DSM-IV: Diagnostic and Statistical Manual of Mental Disorders, 4th Edition
GDS: Geriatric Depression Scale
JPSC-AD: Japan Prospective Studies Collaboration for Aging and Dementia
MCI: Mild cognitive impairment
MMSE: Mini-Mental State Examination
MRI: Magnetic resonance imaging
NINCDS-ADRDA: National Institute of Neurological and Communicative Disorders and Stroke and the Alzheimer’s Disease and Related Disorders Association criteria
NINDS-AIREN: National Institute of Neurological Disorders and Stroke-Association International pour la Recherche et l’Enseignement en Neurosciences criteria
T1WI: Three-dimensional acquisition of T1-weighted images
VaD: Vascular dementia

## References

[1] The World Alzheimer Report (2015). The global impact of dementia.

[2] Asada T (2012). Prevalence of dementia in Japan: past, present and future [in Japanese]. Rinsho Shinkeigaku.

[3] Ninomiya T, Kiyohara Y, Ohara T, Yonemoto K (2015). A study on the future estimation of the elderly population with dementia in Japan [in Japanese].

[4] Ohara T, Hata J, Yoshida D, Mukai N, Nagata M, Iwaki T, Kitazono T, Kanba S, Kiyohara Y, Ninomiya T (2017). Trends in dementia prevalence, incidence, and survival rate in a Japanese community. Neurology..

[5] Livingston G, Sommerlad A, Orgeta V, Costafreda SG, Huntley J, Ames D, Ballard C, Banerjee S, Burns A, Cohen-Mansfield J, Cooper C, Fox N, Gitlin LN, Howard R, Kales HC, Larson EB, Ritchie K, Rockwood K, Sampson EL, Samus Q, Schneider LS, Selbæk G, Teri L, Mukadam N (2017). Dementia prevention, intervention, and care. Lancet..

[6] Ohara T, Ninomiya T, Kubo M, Hirakawa Y, Doi Y, Hata J, Iwaki T, Kanba S, Kiyohara Y (2011). Apolipoprotein genotype for prediction of Alzheimer’s disease in older Japanese: the Hisayama Study. J Am Geriatr Soc.

[7] Harold D, Abraham R, Hollingworth P, Sims R, Gerrish A, Hamshere ML, Pahwa JS, Moskvina V, Dowzell K, Williams A, Jones N, Thomas C, Stretton A, Morgan AR, Lovestone S, Powell J, Proitsi P, Lupton MK, Brayne C, Rubinsztein DC, Gill M, Lawlor B, Lynch A, Morgan K, Brown KS, Passmore PA, Craig D, McGuinness B, Todd S, Holmes C, Mann D, Smith AD, Love S, Kehoe PG, Hardy J, Mead S, Fox N, Rossor M, Collinge J, Maier W, Jessen F, Schürmann B, Heun R, van den Bussche H, Heuser I, Kornhuber J, Wiltfang J, Dichgans M, Frölich L, Hampel H, Hüll M, Rujescu D, Goate AM, Kauwe JS, Cruchaga C, Nowotny P, Morris JC, Mayo K, Sleegers K, Bettens K, Engelborghs S, De Deyn PP, Van Broeckhoven C, Livingston G, Bass NJ, Gurling H, McQuillin A, Gwilliam R, Deloukas P, Al-Chalabi A, Shaw CE, Tsolaki M, Singleton AB, Guerreiro R, Mühleisen TW, Nöthen MM, Moebus S, Jöckel KH, Klopp N, Wichmann HE, Carrasquillo MM, Pankratz VS, Younkin SG, Holmans PA, O'Donovan M, Owen MJ, Williams J (2009). Genome-wide association study identifies variants at CLU and PICALM associated with Alzheimer’s disease. Nat Genet.

[8] Lambert JC, Ibrahim-Verbaas CA, Harold D, Naj AC, Sims R, Bellenguez C, AL DS, Bis JC, Beecham GW, Grenier-Boley B, Russo G, Thorton-Wells TA, Jones N, Smith AV, Chouraki V, Thomas C, Ikram MA, Zelenika D, Vardarajan BN, Kamatani Y, Lin CF, Gerrish A, Schmidt H, Kunkle B, Dunstan ML, Ruiz A, Bihoreau MT, Choi SH, Reitz C, Pasquier F, Cruchaga C, Craig D, Amin N, Berr C, Lopez OL, De Jager PL, Deramecourt V, Johnston JA, Evans D, Lovestone S, Letenneur L, Morón FJ, Rubinsztein DC, Eiriksdottir G, Sleegers K, Goate AM, Fiévet N, Huentelman MW, Gill M, Brown K, Kamboh MI, Keller L, Barberger-Gateau P, McGuiness B, Larson EB, Green R, Myers AJ, Dufouil C, Todd S, Wallon D, Love S, Rogaeva E, Gallacher J, St George-Hyslop P, Clarimon J, Lleo A, Bayer A, Tsuang DW, Yu L, Tsolaki M, Bossù P, Spalletta G, Proitsi P, Collinge J, Sorbi S, Sanchez-Garcia F, Fox NC, Hardy J, Deniz Naranjo MC, Bosco P, Clarke R, Brayne C, Galimberti D, Mancuso M, Matthews F, Moebus S, Mecocci P, Del Zompo M, Maier W, Hampel H, Pilotto A, Bullido M, Panza F, Caffarra P, Nacmias B, Gilbert JR, Mayhaus M, Lannefelt L, Hakonarson H, Pichler S, Carrasquillo MM, Ingelsson M, Beekly D, Alvarez V, Zou F, Valladares O, Younkin SG, Coto E, Hamilton-Nelson KL, Gu W, Razquin C, Pastor P, Mateo I, Owen MJ, Faber KM, Jonsson PV, Combarros O, O'Donovan MC, Cantwell LB, Soininen H, Blacker D, Mead S, Mosley TH, Bennett DA, Harris TB, Fratiglioni L, Holmes C, de Bruijn RF, Passmore P, Montine TJ, Bettens K, Rotter JI, Brice A, Morgan K, Foroud TM, Kukull WA, Hannequin D, Powell JF, Nalls MA, Ritchie K, Lunetta KL, Kauwe JS, Boerwinkle E, Riemenschneider M, Boada M, Hiltuenen M, Martin ER, Schmidt R, Rujescu D, Wang LS, Dartigues JF, Mayeux R, Tzourio C, Hofman A, Nöthen MM, Graff C, Psaty BM, Jones L, Haines JL, Holmans PA, Lathrop M, Pericak-Vance MA, Launer LJ, Farrer LA, van Duijn CM, Van Broeckhoven C, Moskvina V, Seshadri S, Williams J, Schellenberg GD, Amouyel P, European Alzheimer’s Disease Initiative (EADI); Genetic and Environmental Risk in Alzheimer’s Disease; Alzheimer’s Disease Genetic Consortium; Cohorts for Heart and Aging Research in Genomic Epidemiology (2013). Meta-analysis of 74,046 individuals identifies 11 new susceptibility loci for Alzheimer’s disease. Nat Genet.

[9] Shen L, Jia J (2016). An overview of genome-wide association studies in Alzheimer’s disease. Neurosci Bull.

[10] Sheikh JI, Yesavage JA (1986). Geriatric Depression Scale (GDS): recent evidence and development of a shorter version. Clin Gerontol.

[11] Sheehan DV, Lecrubier Y, Sheehan KH, Amorim P, Janavs J, Weiller E, Hergueta T, Baker R, Dunbar GC (1998). The Mini-International Neuropsychiatric Interview (M.I.N.I.): the development and validation of a structured diagnostic psychiatric interview for DSM-IV and ICD-10. J Clin Psychiatry.

[12] American Psychiatric Association (1994). Diagnostic and statistical manual of mental disorders.

[13] Jack CR, Bernstein MA, Fox NC, Thompson P, Alexander G, Harvey D, Borowski B, Britson PJ, Whitwell J, Ward C, Dale AM, Felmlee JP, Gunter JL, Hill DL, Killiany R, Schuff N, Fox-Bosetti S, Lin C, Studholme C, CS DC, Krueger G, Ward HA, Metzger GJ, Scott KT, Mallozzi R, Blezek D, Levy J, Debbins JP, Fleisher AS, Albert M, Green R, Bartzokis G, Glover G, Mugler J, Weiner MW (2008). The Alzheimer’s Disease Neuroimaging Initiative (ADNI): MRI methods. J Magn Reson Imaging.

[14] Fischl B, Salat DH, Busa E, Albert M, Dieterich M, Haselgrove C, van der Kouwe A, Killiany R, Kennedy D, Klaveness S, Montillo A, Makris N, Rosen B, Dale AM (2002). Whole brain segmentation: automated labeling of neuroanatomical structures in the human brain. Neuron.

[15] American Psychiatric Association (1987). Diagnostic and statistical manual of mental disorders.

[16] McKhann G, Drachman D, Folstein M, Katzman R, Price D, Stadlan EM (1984). Clinical diagnosis of Alzheimer’s disease: report of the NINCDS-ADRDA Work Group under the auspices of Department of Health and Human Services Task Force on Alzheimer’s Disease. Neurology.

[17] Román GC, Tatemichi TK, Erkinjuntti T, Cummings JL, Masdeu JC, Garcia JH (1993). Vascular dementia: diagnostic criteria for research studies. Report of the NINDS-AIREN International Workshop. Neurology.

[18] McKeith IG, Boeve BF, Dickson DW, Halliday G, Taylor JP, Weintraub D, Aarsland D, Galvin J, Attems J, Ballard CG, Bayston A, Beach TG, Blanc F, Bohnen N, Bonanni L, Bras J, Brundin P, Burn D, Chen-Plotkin A, Duda JE, El-Agnaf O, Feldman H, Ferman TJ, Ffytche D, Fujishiro H, Galasko D, Goldman JG, Gomperts SN, Graff-Radford NR, Honig LS, Iranzo A, Kantarci K, Kaufer D, Kukull W, VMY L, Leverenz JB, Lewis S, Lippa C, Lunde A, Masellis M, Masliah E, McLean P, Mollenhauer B, Montine TJ, Moreno E, Mori E, Murray M, O'Brien JT, Orimo S, Postuma RB, Ramaswamy S, Ross OA, Salmon DP, Singleton A, Taylor A, Thomas A, Tiraboschi P, Toledo JB, Trojanowski JQ, Tsuang D, Walker Z, Yamada M, Kosaka K (2017). Diagnosis and management of dementia with Lewy bodies: fourth consensus report of the DLB Consortium. Neurology.

[19] Petersen RC, Stevens JC, Ganguli M, Tangalos EG, Cummings JL, DeKosky ST (2001). Practice parameter: early detection of dementia: mild cognitive impairment (an evidence-based review). Report of the Quality Standards Subcommittee of the American Academy of Neurology. Neurology.

[20] Folstein MF, Folstein SE, McHugh PR (1975). “Mini-mental state”. A practical method for grading the cognitive state of patients for the clinician. J Psychiatr Res.

[21] Maeshima S, Osawa A, Maeshima E, Shimamoto Y, Sekiguchi E, Kakishita K, Ozaki F, Moriwaki H (2004). Usefulness of a cube-copying test in outpatients with dementia. Brain Inj.

[22] Procedures manual – ADNI: https://adni.loni.usc.edu/wp-content/uploads/2008/07/adni2-procedures-manual.pdf. Accessed 10 June 2020.

[23] Mamiya Y, Nishio Y, Watanabe H, Yokoi K, Uchiyama M, Baba T, Iizuka O, Kanno S, Kamimura N, Kazui H, Hashimoto M, Ikeda M, Takeshita C, Shimomura T, Mori E (2016). The Pareidolia test: a simple neuropsychological test measuring visual hallucination-like illusions. PLoS One.

[24] Dacks PA, Andrieu S, Blacker D, Carman AJ, Green AM, Grodstein F, Henderson VW, James BD, Lane RF, Lau J, Lin PJ, Reeves BC, Shah RC, Vellas B, Yaffe K, Yurko-Mauro K, Shineman DW, Bennett DA, Fillit HM (2014). Dementia prevention: optimizing the use of observational data for personal, clinical, and public health decision-making. J Prev Alzheimers Dis.

[25] Noguchi-Shinohara M, Yuki S, Dohmoto C, Ikeda Y, Samuraki M, Iwasa K, Yokogawa M, Asai K, Komai K, Nakamura H, Yamada M (2013). Differences in the prevalence of dementia and mild cognitive impairment and cognitive functions between early and delayed responders in a community-based study of the elderly. J Alzheimers Dis.

[26] Yamada M, Sasaki H, Mimori Y, Kasagi F, Sudoh S, Ikeda J, Hosoda Y, Nakamura S, Kodama K (1999). Prevalence and risks of dementia in the Japanese population: RERF's adult health study Hiroshima subjects. Radiation Effects Research Foundation. J Am Geriatr Soc.

[27] Ikeda M, Hokoishi K, Maki N, Nebu A, Tachibana N, Komori K, Shigenobu K, Fukuhara R, Tanabe H (2001). Increased prevalence of vascular dementia in Japan: a community-based epidemiological study. Neurology.

[28] Wada-Isoe K, Uemura Y, Nakashita S, Yamawaki M, Tanaka K, Yamamoto M, Shimokata H, Nakashima K (2012). Prevalence of dementia and mild cognitive impairment in the rural island town of Ama-cho, Japan. Dement Geriatr Cogn Dis Extra.

[29] Imrey PB (2020). Limitations of meta-analyses of studies with high heterogeneity. JAMA Netw Open.

[30] Lee Y, Shinkai S (2005). Correlates of cognitive impairment and depressive symptoms among older adults in Korea and Japan. Int J Geriatr Psychiatry.

[31] Wada T, Ishine M, Sakagami T, Okumiya K, Fujisawa M, Murakami S, Otsuka K, Yano S, Kita T, Matsubayashi K (2004). Depression in Japanese community-dwelling elderly--prevalence and association with ADL and QOL. Arch Gerontol Geriatr.

