# Supplementary material for: Study design and baseline characteristics of a population-based prospective cohort study of dementia in Japan: the Japan Prospective Studies Collaboration for Aging and Dementia (JPSC-AD)
Source: Environ Health Prev Med. 2020 Oct 31;25:64. doi: 10.1186/s12199-020-00903-3 (PMC7603740; doi:10.1186/s12199-020-00903-3)
Supplement: Supplementary file 1 — Additional file 1: Table S1. Instruments to measure blood pressure, electrocardiogram, and handgrip strength. Table S2. Measurement methods by item. Table S3. The baseline characteristics for 5,257 subjects at three research sites (Nakajima Town, Nakayama Town, and Hisayama Town). Table S4. Age-specific prevalence of dementia at baseline among 5,257 subjects at three research sites (Nakajima Town, Nakayama Town, and Hisayama Towna); participation rate: 85%), where surveys by home visit or visits to nursing homes or other facilities (e.g., long-stay hospitals) were conducted in addition to the surveys at the health centres or hospital. Figure S1. Organization of the Japan Prospective Studies Collaboration for Aging and Dementia (JPSC-AD). [file 12199_2020_903_MOESM1_ESM.pdf]

Supplementary Table 1: Instruments to measure blood pressure, electrocardiogram, and handgrip strength

Parameter	Research site	Mearuing instrument	Manufacturing company
Blood pressure	Hirosaki City	ES-P2000BR	Terumo Corporation, Tokyo, Japan
	Yahaba Town	HBP-9020	Omron Healthcare, Kyoto, Japan
	Nakajima Town	HEM-8712	Omron Healthcare, Kyoto, Japan
	Arakawa Ward	BP-103iⅡ	Fukuda Colin, Tokyo, Japan
	Ama Town	H55	Terumo Corporation, Tokyo, Japan
	Nakayama Town	HEM-8713	Omron Healthcare, Kyoto, Japan
	Hisayama Town	HBP-9020	Omron Healthcare, Kyoto, Japan
	Arao City	TM-2656VPW	A&D Company, Tokyo, Japan
Electrocardiogram	Hirosaki City	ECG-1350	Nihon Kohden, Tokyo, Japan
	Yahaba Town	FCP-8800	Fukuda Denshi, Tokyo, Japan
	Nakajima Town	FCP-8221	Fukuda Denshi, Tokyo, Japan
	Arakawa Ward	FCP-8321	Fukuda Denshi, Tokyo, Japan
	Ama Town	FCP-8221	Fukuda Denshi, Tokyo, Japan
	Nakayama Town	FCP-8221	Fukuda Denshi, Tokyo, Japan
	Hisayama Town	FCP-7541	Fukuda Denshi, Tokyo, Japan
	Arao City	FCP-7431	Fukuda Denshi, Tokyo, Japan
Handgrip strength	Hirosaki City	T.K.K 5001	Takei Scientific Instruments, Niigata, Japan
	Yahaba Town	T.K.K.5401	Takei Scientific Instruments, Niigata, Japan
	Nakajima Town	T.K.K.5401	Takei Scientific Instruments, Niigata, Japan
	Arakawa Ward	YS	Tsutsumi, Tokyo, Japan
	Ama Town	T.K.K.5401	Takei Scientific Instruments, Niigata, Japan
	Nakayama Town	T.K.K.5401	Takei Scientific Instruments, Niigata, Japan
		261-006-05YX	Muranaka Medical Instruments, Osaka, Japan
	Hisayama Town	T.K.K.5401	Takei Scientific Instruments, Niigata, Japan
	Arao City	T-2177	TOEI LIGHT, Saitama, Japan

Supplementary Table 2: Measurement methods by item

Measurement item	Measuring method
**Blood test**	
White blood cells	Flow cytometric method
Red blood cells	Bioelectrical impedance method
Hemoglobin	Sodium lauryl sulfate-hemoglobin method
Hematocrit	Red blood cell pulse height detection method
Platelet	Bioelectrical impedance method
Total protein	Biuret method
Albumin	Modified bromocresol purple method
Urea nitrogen	Urease and leucine dehydrogenase method
Creatinine	Enzymatic method
Uric acid	Enzymatic method
Total bilirubin	Enzymatic method
Aspartate transaminase	JSCC reference method
Alanine transaminase	JSCC reference method
Alkaline phosphatase	JSCC reference method
Lactate dehydrogenase	JSCC reference method
γ-glutamyl transferase	JSCC reference method
Total cholesterol	Enzymatic method
Serum HDL cholesterol	Enzymatic method
Serum LDL cholesterol	Enzymatic method
Triglycerides	Enzymatic method
Glucose	Enzymatic method
Hemoglobin A1c	Enzymatic method (NGSP)
Glycated albumin	Enzymatic method
Insulin	Chemiluminescent immunoassay
Sodium	Electrode method
Potassium	Electrode method
High-sensitivity CRP	Nephelometric method
Free thyroxine (T_4)	Chemiluminescent immunoassay
Thyroid-stimulating hormone	Chemiluminescent immunoassay
**Urine test**	
Urinary albumin	Turbidimetric immunoassay
Urinary creatinine	Enzymatic method

Abbreviations: CRP, C-reactive protein; HDL, high-density lipoprotein; JSCC, Japan Society of Clinical Chemistry; LDL, low-density lipoprotein.

Supplementary Table 3: The baseline characteristics for 5,257 subjects at three research sites (Nakajima Town, Nakayama Town, and Hisayama Town)

Variables	Mean, median, or frequency [a)]	% of missing value
**Demographic factors and lifestyles**		
Age, years	76.7 (8.2)	0.0
Men, %	42.0	0.0
Education≤9 years, %	46.6	11.0
Current smoker, %	36.0	10.1
Current drinker, %	7.8	10.1
Regular exercise[b], %	31.8	24.0
**Physical examinations**		
Systolic blood pressure, mmHg	136.1 (19.2)	25.0
Diastolic blood pressure, mmHg	75.8 (12.1)	25.0
BMI, kg/m^2	23.3 (3.5)	23.3
Waist circumference (at umbilicus), cm	85.4 (9.7)	29.8
Electrocardiogram abnormalities[c], %	17.5	32.9
**Comorbidities and medications**		
History of cardiovascular disease[d], %	15.1	6.5
History of cancer, %	13.5	6.4
Hypertension [e)], %	71.2	9.8
Diabetes mellitus[f], %	23.2	27.6
Use of antihypertensive agents, %	55.3	8.2
Use of glucose-lowering agents (including insulin therapy), %	15.0	8.4
Use of lipid-modifying agents, %	32.0	8.7
**Blood test**		
White blood cells, x10^9/ L	5.6 (1.6)	29.0
Red blood cells, x10^{12}/ L	4.3 (0.5)	29.0
Hemoglobin, g/L	134.7 (15.7)	29.0
Hematocrit, %	41.1 (4.5)	29.0
Platelet, x10^9/ L	227.1 (67.9)	29.0
Serum total protein, g/L	73.2 (4.7)	31.1
Serum albumin, g/L	42.3 (3.2)	31.1
Serum urea nitrogen, mmol/L	6.2 (2.0)	31.1
Serum creatinine, µmol/L	65.4 (55.7-79.6)	31.1
Estimated GFR [g)], ml/min/1.73m^2	65.7 (12.8)	31.1
Serum uric acid, µmol/L	309.3 (81.0)	31.1
Serum total bilirubin, µmol/L	13.7 (10.3-15.4)	31.1
Serum aspartate transaminase, U/L	23 (20-28)	31.1
Serum alanine transaminase, U/L	17 (13-23)	31.1
Serum alkaline phosphatase, U/L	232 (192-281)	31.1
Serum lactate dehydrogenase, U/L	203 (181-227)	31.1

Serum γ-glutamyl transferase, U/L	22 (16-35)	31.1
Serum total cholesterol, mmol/L	5.1 (0.9)	31.1
Serum LDL cholesterol, mmol/L	2.9 (0.8)	31.1
Serum HDL cholesterol, mmol/L	1.5 (0.4)	31.1
Fasting serum triglycerides, mmol/L	1.1 (0.8-1.5)	61.0
Fasting blood glucose, mmol/L	5.3 (1.2)	61.0
Hemoglobin A1c (NGSP), %	5.8 (0.7)	31.1
Serum glycated albumin, %	15.8 (2.9)	33.9
Serum sodium, mmol/L	141.6 (2.3)	31.4
Serum potassium, mmol /L	4.5 (0.7)	31.1
Serum high-sensitivity CRP, mg/L	0.48 (0.23-1.03)	31.2
Serum free thyroxine (T_4), pmol/L	14.6 (2.2)	31.2
Serum thyroid stimulating hormone, µIU/mL	1.90 (1.27-2.88)	31.2
**Urine test**		
Proteinuria (dipstick ≥1+), %	9.1	34.5
Urinary albumin:creatinine ratio, mg/gCr	11.6 (6-29.3)	35.5
**ADL and Sleep**		
ADL disability (Barthel Index ≤95), %	19.9	2.8
Functional capacity impairment (TMIG-IC ≤12), %	53.1	10.1
Maximal handgrip strength, kg	25.8 (9.1)	18.6
Usual speed 5-m walking time, sec	4.5 (1.8)	39.0
**Cognitive function**		
Mini-Mental State Examination, points	28 (25-29)	14.3
**Depressive status**		
Geriatric depression scale, points	2 (1-4)	17.1
Depressive symptoms (Geriatric depression scale ≥6 points), %	17.6	17.1
Depression, %	1.4	0.0

Abbreviations: ADL, activities of daily living; BMI, body mass index; CRP, C-reactive protein; HDL, high density lipoprotein; LDL, low-density lipoprotein; NGSP, National Glycohemoglobin Standardization Program; TMIG-IC, Tokyo Metropolitan Institute of Gerontology Index of Competence; Uprot, urinary protein concentration.

a) Values are shown as the mean (standard deviation), median (interquartile range), or frequency, as appropriate. Subjects with missing values were excluded from the analysis for each relevant variable.

b) Regular exercise was defined as any physical activity performed for at least 30 min twice per week over the most recent year or longer.

c) Electrocardiogram abnormalities were defined as the presence of left ventricular hypertrophy, ST depression, and/or atrial fibrillation.

d) Cardiovascular disease was defined as stroke, coronary heart disease and/or coronary intervention.

e) Hypertension was defined as blood pressure ≥140/90 mmHg and/or use of antihypertensive agents.

f) Diabetes was defined as fasting blood glucose ≥126 mg/dL, casual blood glucose ≥200 mg/dL, hemoglobin A1c ≥6.5%, and/or glucose-lowering agents.

g) Estimated GFR was calculated by using the Japanese coefficient modified CKD-EPI (Chronic Kidney Disease Epidemiology Collaboration) equation.

Note: Conversion factors for units are shown at the footnote in Table 1.

Supplementary Table 4: Age-specific prevalence of dementia at baseline among 5,257 subjects at three research sites (Nakajima Town, Nakayama Town, and Hisayama Town[a)]; participation rate: 85%), where surveys by home visit or visits to nursing homes or other facilities (e.g., long-stay hospitals) were conducted in addition to the surveys at the health centres or hospital.

Age group, years	Number of subjects	Number of dementia	Prevalence of dementia
**Overall**			
65-69	1,314	19	1.4%
70-74	1,049	37	3.5%
75-79	992	101	10.2%
80-84	862	180	20.9%
85-89	639	272	42.6%
≥90	401	251	62.6%
**Total**	5,257	860	16.4%
**Men**			
65-69	628	9	1.4%
70-74	493	16	3.2%
75-79	416	40	9.6%
80-84	355	67	18.9%
85-89	215	73	34.0%
≥90	101	43	42.6%
**Total**	2,208	248	11.2%
**Women**			
65-69	686	10	1.5%
70-74	556	21	3.8%
75-79	576	61	10.6%
80-84	507	113	22.3%
85-89	424	199	46.9%
≥90	300	208	69.3%
**Total**	3049	612	20.1%

a) In Hisayama Town, surveys by home visit and visits to nursing homes or other facilities were conducted for 488 residents (184 men and 304 women) in addition to 1,714 participants of the JPSC-AD study.

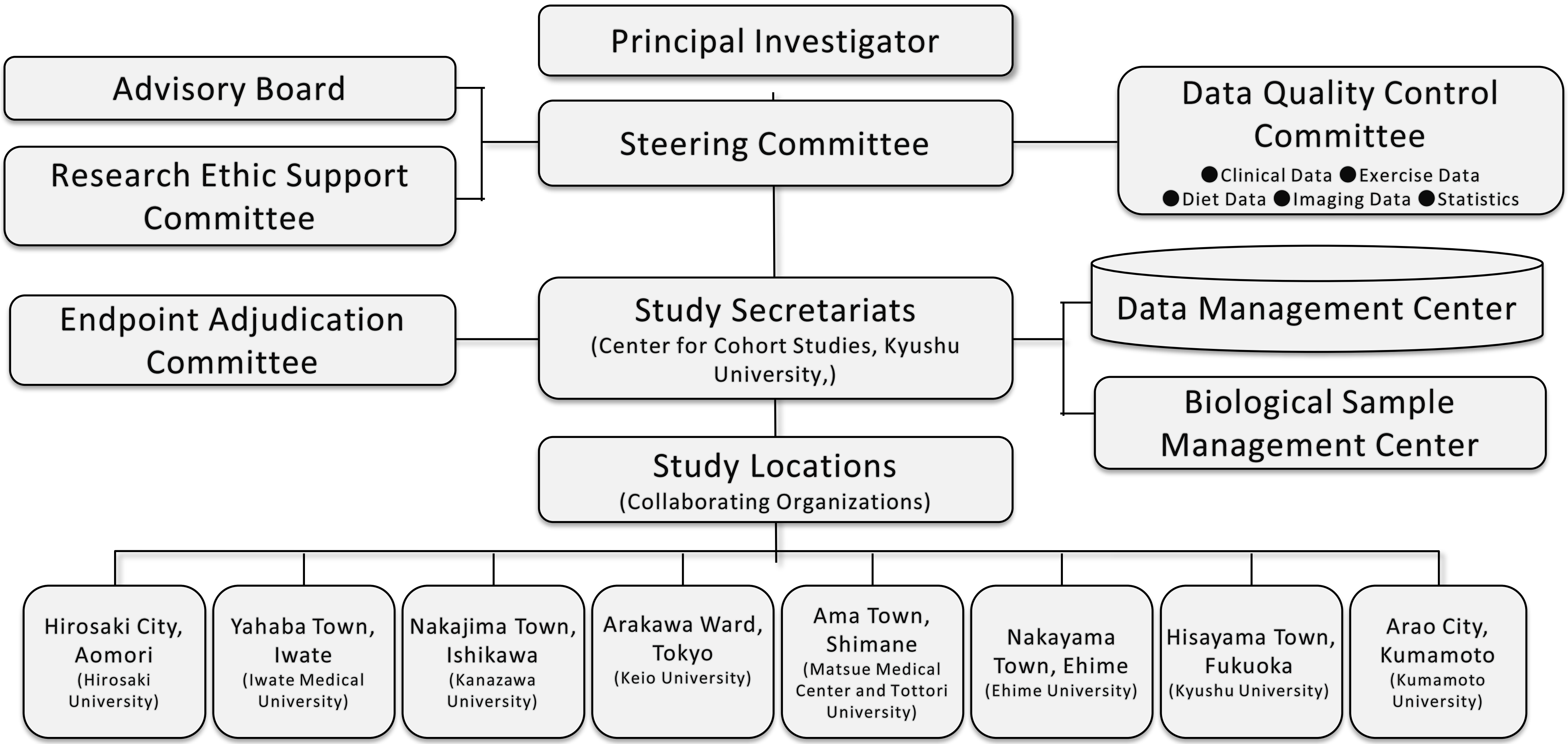

Supplementary Figure 1: Organization of the Japan Prospective Studies Collaboration for Aging and Dementia (JPSC-AD)
